# 65 Years on—Aflatoxin Biomarkers Blossoming: Whither Next?

**DOI:** 10.3390/toxins16110496

**Published:** 2024-11-18

**Authors:** Thomas W. Kensler, David L. Eaton

**Affiliations:** 1 Department of Environmental Health and Engineering, Johns Hopkins Bloomberg School of Public Health, Baltimore, MD 21205, USA; 2 Department of Environmental and Occupational Health Sciences, School of Public Health, University of Washington, Seattle, WA 98195, USA

**Keywords:** aflatoxin B_1_, aflatoxin–N^7^–guanine, aflatoxin–albumin adduct, biomarkers, validation, exposome, liver cancer

## Abstract

Aflatoxins are mycotoxins produced by *Aspergillus flavus* and several other related organisms and are common contaminants of numerous grains and nuts, especially maize (corn) and peanuts. Although, undoubtedly, aflatoxins have been present in the food of humans for millennia, their toxic effects were not discovered until 1960, first becoming evident as a non-infectious outbreak of poisoning of turkeys (Turkey X disease) arising from contaminated groundnut meal. The elucidation of specific chemical structures in 1963 led to the rapid characterization of aflatoxins as among the most potent chemical carcinogens of natural origin ever discovered. As a frontispiece to the Special Issue “65 Years on from Aflatoxin Discovery—A Themed Issue in Honor of Professor John D. Groopman”, we highlight many of Professor Groopman’s important contributions utilizing urinary (aflatoxin–N^7^–guanine) and, especially, serum (aflatoxin–albumin adducts) biomarkers; this work focused on over 40+ years of the development of analytical methods to measure biomarkers of aflatoxin exposure and their application in experimental and clinical studies. Collectively, this work serves as a template for using chemical-specific biomarkers as key tools to probe ‘exposure–disease relationships’—in this instance, dietary aflatoxins and liver cancer. New approaches to measuring carcinogen biomarkers will build upon this ‘aflatoxin paradigm’ to inform the public health implications of diverse exposures around the world.

## 1. Introduction

Aflatoxins derive their name from their fungal origin: *Aspergillus flavus* toxin. Although these mycotoxins have undoubtedly been around for millennia, they were not discovered until ~1960, when an outbreak of hepatotoxicity killed over 100,000 turkeys in England, dubbed ‘Turkey X disease [1,2,3]. The origin of the poisonings was traced to Brazilian groundnut meal (“Rosetta meal”), and, from there, the *Aspergillus flavus* mold was identified as the likely culprit. Soon after, the toxic effects of the Brazilian groundnut meal were confirmed in numerous other species, including ducklings, chicken, young pheasants, cattle, rats, and pigs. Necropsies of some of the animals identified the liver as the primary organ of toxicity, including evidence of liver cancer development (see [2,4] for excellent reviews of the history of aflatoxin discovery).

The characterization of the toxic entity produced by *A. flavus* was described in 1962. The initial extract had a blue fluorescence—thus, the moniker “Aflatoxin B”. A second material separated by thin-layer chromatography had a green fluorescence—thus, the moniker for aflatoxin G [5]. Subsequent work identified the structures of both aflatoxin B and aflatoxin G in 1963 [6,7]. 

The outbreak of poisonings among turkeys was characteristic of the acute or sub-acute toxicity of aflatoxin, with typical histopathological findings that include enlarged and mottled livers with periportal necrosis, hemorrhaging, and the accumulation of fat [8]. The acute/sub-acute toxicity of aflatoxins is relatively high (<20 mg/kg) in most vertebrate species tested but can vary substantially among species. Ducks, trout, and rabbits tend to be the most sensitive, with LD_50_ values less than 1 mg/kg, whereas the chicken and Porton rat exhibit the highest LD_50_ of ~18 mg/kg [4]. Acute toxicity, however, is not a good predictor of relative species sensitivity to the carcinogenic effects of aflatoxin, as discussed in more detail in the papers in this Special Issue. 

Although the evident toxic effects of *A. flavus*-contaminated diets were largely associated with acute and sub-acute hepatotoxicity, the discovery of the potent carcinogenic effects of aflatoxin B_1_ [AFB_1_] followed quickly. The first reports of the evidence of the carcinogenicity of the toxic components of groundnut meal was reported in 1961 [9]. In 1958–1959, liver tumors were identified in guinea pigs given a ‘routine’, standardized Medical Research Council (MRC) diet containing 15% groundnut meal. However, the etiologic factor(s) in the diet that caused the liver tumors was unknown. In 1960–1961, ‘Turkey X disease’ stimulated numerous studies to identify the causal agents within the groundnut meal. The association between the groundnut meal that caused Turkey X disease and the fact that groundnut meal was a constituent in the guinea pig diet led Schoental [9] to deduce that fungal contaminants in the groundnut meal were carcinogenic. Several subsequent experimental studies demonstrated the potent hepatocarcinogenic effects of the major isolate, AFB_1_ [10,11,12]. Barnes and Butler [11] carried out what was perhaps the first ‘controlled study’ in rats of aflatoxin carcinogenesis. The availability of reasonably pure AFB_1_ was limiting for such studies. A summary of the study illustrates well the challenges of conducting such studies: “*Thus 3/3 rats receiving a diet containing 1·75 p.p.m. aflatoxin for 89 days ultimately developed liver cancer after a further 300 or more days on a diet that produces no liver changes in control rats. The rats ate on average 16 g of the food containing aflatoxin each day, so that it is possible to calculate that the carcinogenic dose of aflatoxin for rats is certainly not greater than 2·5 mg per rat*”. Although the sample size was small and the duration of exposure was limited, this study clearly illustrated the strong hepatocarcinogenic potential of AFB_1_. Another study with ducks reported in 1965 [13] also demonstrated the potent hepatotoxic and hepatoxic carcinogenic effects of AFB_1_ in groundnut meal. 

In 1967, Gerald Wogan and Paul Newberne at MIT conducted the first controlled, dose–response study of AFB_1_ administered in the diet for up to 80 weeks in rats [14]. Although previous studies had demonstrated the potential toxicity and hepatocarcinogenic effects of extracts of *A. flavus*-contaminated groundnut meal [12,15,16], this was the first study that directly utilized purified AFB_1_ with relatively long-term exposures of 60–80 weeks. Multiple different studies and design protocols were used, with several to assess acute as well as chronic toxicity. Perhaps the most informative study involved the administration of 15, 300, or 1000 ppb AFB_1_ daily for 60 weeks or longer. The study demonstrated the remarkable potency of AFB_1_ following prolonged feeding to both male and female Fischer rats. Even at the lowest dose of 15 ppb in the diet, 100% of both male (12/12 at 68 weeks) and female (13/13 at 80 weeks) rats had developed hepatocellular carcinomas.

**Figure 1:**
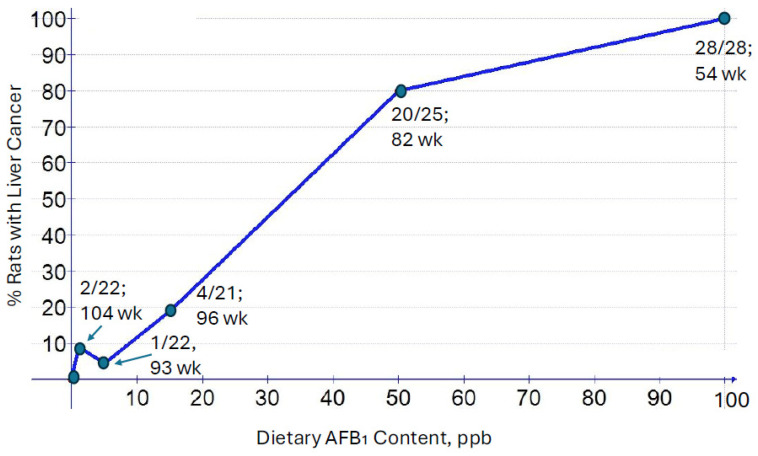
Dose–response relationship between AFB_1_ dose and malignant liver tumors in male rats. The numbers of animals with tumors out of the total number of animals is shown for each dose point. Also shown are the first ‘time to tumor’ data for each dose point. See Wogan et al., 1974 for details [17].

Based on the remarkable potency of AFB_1_ from this initial study, Wogan et al. [17], in 1974, conducted a lifetime carcinogenicity study using male Fischer rats fed with five different doses incorporated into the diet, ranging from 1 to 100 ppm, plus a control group. The results are shown in Figure 1.

As with the initial study, there were some suggestions that a dose as low as 1 ppb in the diet was sufficient to induce hepatocellular carcinomas (HCCs) when given over the lifetime of the animals. All 28 rats given 100 ppb developed HCCs, with the first carcinoma-related fatality occurring at 54 weeks at that dose. At a concentration of only 15 ppb, which is below the current FDA action limit (20 ppb) for aflatoxin (the sum of the B- and G-series) in food in the U.S., nearly 20% of the animals developed liver cancer by the end of the two-year study. Based on these results, and numerous other studies in other species, aflatoxin appears to be the most potent genotoxic carcinogen of natural origin ever identified. The molecule referred to commonly as ‘dioxin’ (2,3,7,8-tetrachlorodibenzo-p-dioxin; TCDD) has a TD50 (see the Lhasa Carcinogen Potency Database, that also includes TD50 data from the Ames and Gold Carcinogen Database: https://lcdb.lhasacloud.org/study-information/44411599 accessed on 11 November 2024)) (estimated dose where 50% of exposed animals would develop cancer) about 15-times lower (more potent) than that for AFB_1_ (0.00014 μg/kg-d for TCDD, versus 0.0020 μg/kg-d for AFB_1_, using the Ames and Gold Carcinogen Potency Database). The mode of action for the hepatocarcinogenic effects of TCDD is via a receptor-mediated enhancement of a signaling pathway (Aryl hydrocarbon Receptor, or AhR) that promotes tumorigenesis, rather than genotoxic events that initiate carcinogenesis. A more appropriate comparison of carcinogenic potency would be with the nitrosamine, N-nitroso-dimethylamine (NDMA). Like AFB_1_, NDMA is a potent genotoxic hepatocarcinogen. The TD50 for NDMA in rats is 0.09 μg/kg-d, or about 45-times less potent than AFB_1_. The FDA acceptable intake for pharmaceuticals contaminated with NDMA is 96 ng. Using the TD50 values for a relative potency comparison, that would equate to an acceptable daily intake of AFB_1_ of about 2 ng. If a commodity (say, corn or peanuts) is contaminated with AFB_1_ at 15 ppb (15 μg/kg), which is below the FDA action level of 20 ppb, a one ounce (28 g) serving would contain 420 ng of AFB_1_, which is more than 200-times the potential carcinogenic dose of a pharmaceutical containing the FDA allowable limit of NDMA. Of course, this exercise assumes that the potency for both NDMA and AFB_1_ determined in rats is similar in humans. As discussed in detail by Eaton et al. in this series, there are important differences among species in how AFB_1_ is metabolized that largely determine the relative carcinogenic potency across species. There are also important species differences in the carcinogenicity of NDMA between humans and rats [18]. The point here is that it was known in the 1960s that AFB_1_ was a very potent carcinogen in several species and, thus, human studies were of paramount importance to determining the public health significance of dietary contamination with AFB_1_. One of the biggest challenges in designing such epidemiology studies is assessing the extent of exposure, or dose. The use of biological markers (biomarkers) has thus played a critical role in the design and conducting of many epidemiology studies over the past 4+ decades that have established AFB_1_ as a potent human liver carcinogen. The contributions of Professor John Groopman in the development of biomarkers of AFB_1_ exposure were essential for these studies. 

## 2. Identification and Development of Aflatoxin Biomarkers of Internal Dose and Effective Dose

**Figure 2:**
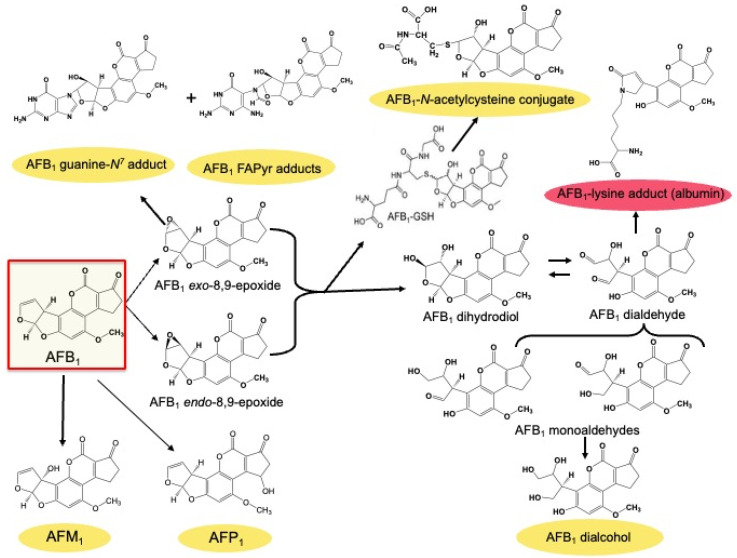
AFB_1_ metabolites used as biomarkers from biofluids. Various cytochromes, P450, form oxidative metabolites, of varying toxicity. AFB-8,9-oxide is highly toxic and responsible for most, if not all, of the toxic and carcinogenic effects of AFB_1_. However, it can be detoxified by conjugation with glutathione, via specific glutathione *S*-transferases. Formation of DNA and protein adducts provide stable urinary and serum biomarkers of exposure with biological half-lives of ~8 h and ~30 days, respectively. Yellow: urine; red: serum or plasma.

***Urinary Aflatoxin Metabolites***: As discussed in a review elsewhere in this Special Issue (Eaton et al.), the metabolism of AFB_1_ has been well-characterized in multiple species including humans. Figure 2 summarizes the oxidative, conjugation, and adduct products of AFB_1_ metabolism that have been used as biomarkers of the internal dose and, sometimes, early biological effect. In some instances, they can be quantitatively linked to estimates of dietary exposure.

**Figure 3:**
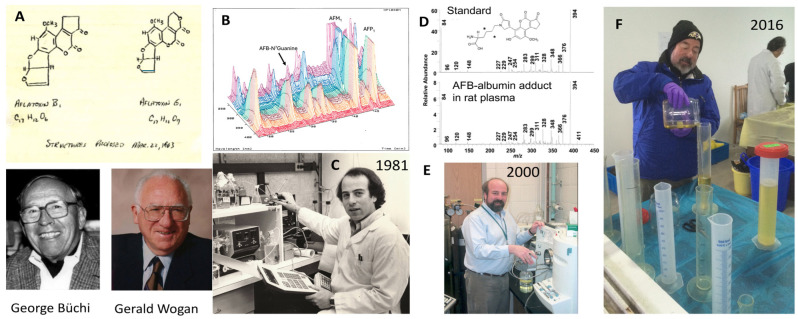
Pioneers of AFB_1_ discovery and biomarker development. (**A**) Proposed structures of AFB_1_ and AFG_1_ by George Büchi on 22 March 1963 and reported later that year by Büchi, Wogan and colleagues [6]. Professor Gerald Wogan was the doctoral mentor for Prof. Groopman. Courtesy of Prof John Essigmann, MIT. (**B**) Initial chromatography of aflatoxin–N^7^–guanine by HPLC and photodiode array detection in human urine in 1987 by Groopman. (**C**) Dr Groopman with his first HPLC (1981). Courtesy of Prof. John Groopman. (**D**) Mass spectrometry of aflatoxin–lysine adduct standard and in rat serum. * Indicates position of stable isotope labels for internal standard. (**E**) John Groopman with his first mass spectrometer for biomarker quantification, a Thermo LCQ (2000). Photo courtesy of Thomas Kensler. (**F**) John Groopman measuring volumes of urine in samples collected during molecular epidemiology studies and chemoprevention clinical trials in Qidong, China, an endemic area for aflatoxin exposure, with a high incidence of liver cancer (2016). Photo courtesy of Thomas Kensler.

Several metabolites, notably, aflatoxin M_1_ (AFM_1_) along with aflatoxin-8,9-epoxide-derived adducts with DNA, RNA, and protein, were used for the initial monitoring of human exposures. These studies used urine as the biospecimen source due its role as a major portal for excretion, the ease of collection, and the simplicity of processing for analyses. However, the short whole-body half-lives of these metabolites, coupled with typically heterogenous exposures, renders evaluations of the associations of individual levels with the risk of disease challenging. Studies of aflatoxin biomarkers in human populations began in the Philippines, where investigators first demonstrated that an oxidative metabolite of AFB_1_, AFM_1_, could be measured in urine as an internal dose marker for people consuming aflatoxin-contaminated peanut butter [19]. Studies in two areas with a high incidence of liver cancer, the People’s Republic of China and The Gambia, West Africa, reported that urinary levels of oxidative aflatoxin metabolites followed a dose-dependent relationship with aflatoxin ingestion for AFM_1_ but not for aflatoxin P_1_ (AFP_1_), another oxidative metabolite [20]. From initial measures using thin-layer chromatography and fluorescence detection to the use of immunoaffinity antibodies for sample enrichment coupled with high-performance liquid chromatography with spectrometric or fluorescence detectors to mass spectrometry, the methods for quantitating urinary biomarkers have improved vastly the sensitivity and specificity of such measures. Professor John Groopman contributed greatly to these advancements [20,21,22,23,24,25,26] (Figure 3, panels B, C, E, and F).

While oxidative metabolites reflect the internal dose, aflatoxin–DNA adducts excreted in urine following either spontaneous depurination or excision repair offer direct insights into the ultimately carcinogenic dose, given the potent genotoxicity of aflatoxin–DNA damage products that are critical to the carcinogenic properties of AFB_1_. The ultimate carcinogenic form of AFB_1_, the 8,9-epoxide (originally termed the 2,3-oxide), was described in the mid-1970s [27] and determined to mediate the formation of the principal AFB_1_–DNA adduct formed in vivo in rat liver [28,29,30] and later found to be excreted in rat urine as well [31]. Validation studies in rats reported a linear relationship between AFB_1_ dose and the excretion of the aflatoxin–N^7^–guanine adduct in urine over the initial 24 h period [32]. Subsequent biomonitoring studies in The Gambia and The Peoples’ Republic of China demonstrated the feasibility of measuring this DNA damage biomarker in urine from humans exposed to aflatoxins and further characterized its kinetic features [33,34]. While the classic dose–response is a critical element for biomarker performance, the modulation of internal dose, in addition to the external or administered dose, is a valuable tool for validation. Chemopreventive interventions that reduce the absorption of AFB_1_ from the gastrointestinal tract (e.g., chlorophyllin) or alter hepatic metabolism to block epoxide formation or enhance its detoxication through conjugation with glutathione (e.g., ethoxyquin, oltipraz, and 2-cyano-3,12-dioxooleana-1,9(11)-dien-28-imidazolide [CDDO-Im]) confirm this element of biomarker modulability. In rats, the decreased formation of aflatoxin–N^7^–guanine adducts in liver are associated with a reduction in tumor burden following chemopreventive interventions with the agents ethoxyquin, oltipraz, and CDDO-Im [35,36,37]. 

Chlorophyllin, a water-soluble derivative of chlorophyll, inhibits liver cancer development in aflatoxin-treated trout [38], wherein the primary mode of action is thought to be the sequestration of aflatoxin by chlorophyllin by forming a non-covalent complex in a 1:1 stacking ratio [39]. In 1997, a randomized, double-blind, placebo-controlled chemoprevention trial with chlorophyllin was conducted in Qidong, China. The ingestion of chlorophyllin tablets (100 mg) at each meal led to an overall 55% reduction in median urinary levels of aflatoxin–N^7^–guanine adducts collected 3 months into the intervention compared with those subjects taking a placebo [40]. An earlier clinical trial in this aflatoxin-endemic area with oltipraz led to the diminished excretion of AFM_1_ and increased excretion of aflatoxin–*N*–acetylcysteine in urine [41], suggesting a likely reduction of DNA damage in the individuals receiving the repurposed drug compared to a placebo. 

***Serum Aflatoxin–Albumin Adducts***: Day-to-day aflatoxin exposure through the diet can be very heterogeneous, reflecting the varied contamination within dietary staples such as ground nuts, maize, and grains and across growing and storage seasons. Consequently, biomarkers with short whole-body half-lives (~8 h), such as AFM_1_, aflatoxin–*N*–acetylcysteine, or aflatoxin–N^7^–guanine excreted in urine reflect only the most recent of exposures. Longer half-life biomarkers offer a promise of more integrated exposure assessments, smoothing the day-to-day variability to better reflect a steady-state level of exposure. Towards that end, aflatoxin–albumin adducts measured in serum samples have been used widely in animal studies as well as human ecological surveys and interventional trials. Importantly, they have been used to understand dose–response relationships between exposures and health outcomes, both acute toxicities and chronic manifestations including liver cancer. The biological half-life of aflatoxin–albumin adducts in serum is estimated to be about 30 days; in rats, it is much shorter (2–3 days).

Albumin is now recognized as a reservoir for binding many xenobiotics and endobiotics, especially at the cysteine in position 34. However, several other amino acids serve as targets for adduct formation [42]. The major aflatoxin–albumin adduct forms at a lysine residue. Sabbioni and colleagues [43] initially described the properties of the major serum albumin adduct formed by AFB_1_ in vivo in rats. Subsequently, they examined the relationships between dose (dietary exposure), yield, and steady-state levels in humans. A split bowl assessment of aflatoxin in foods eaten by study participants and the measurement of serum albumin adducts examined the dose relationship between ingestion and biomarker levels over a one-week period [44]. Regression analysis indicated a highly significant association between the amount of aflatoxin ingested and biomarker levels. An average aflatoxin–lysine adduct yield of 0.38 ng aflatoxin–lysine/µg AFB_1_ from the diet was proposed. No differences by gender were observed. Similar values have been estimated from dosimetry experiments in rats [43]. Despite the differences in half-lives of the serum and urinary aflatoxin biomarkers, the levels of both aflatoxin–albumin adducts and/or aflatoxin–N^7^–guanine adducts have been shown to correlate with aflatoxin exposure [33,34,45].

## 3. Validation of Aflatoxin Biomarkers: Are They Risk Markers Too? 

The validation of aflatoxin metabolites and albumin adducts as exposure biomarkers has been accomplished successfully [46,47]. Beginning with the recognition of AFB_1_ as a hepatocarcinogen in trout and rats, and, as a suspected human carcinogen, the development of rigorous methodologies for sensitive and selective measures of biomarkers in biofluids occurred in lockstep with general analytical advancements. The current levels of detection are more than suitable for biomonitoring in human populations. Animal studies determined the dose–response and temporal relationships to exposures. Cross-sectional studies of these biomarkers in human populations with high exposures to aflatoxins confirmed the major elements of the rodent studies. Longitudinal studies, albeit few, have noted that the tracking of repeated measures over months to years is low, unlike measures of blood pressure or blood cholesterol in individuals as examples. This outcome suggests that, while population-based monitoring may be robust, linking individual levels to health outcomes may be an overreach. The long-term stability of the aflatoxin analytes stored in frozen urine and serum have also been confirmed [48]. Confidence in the interpretation of aflatoxin biomarker levels, even at the population level, required additional steps of inquiry. Once more, animal models were critical in defining linkages between biomarkers and health outcomes, typically hepatocarcinogenesis. Most informative have been case–control, cohort, and interventional studies in humans.

***Animal studies***. Several studies in rats have directly examined the predictive value of aflatoxin biomarkers in the subsequent tumor burden (presumptive, preneoplastic lesions such as GGT+ or GSTP+ foci) or the lifetime incidence of hepatocarcinogenesis. These were two-armed studies examining the chemopreventive efficacy of agents now known to be inducers of the nuclear factor erythroid 2–related factor 2 (NRF2) cytoprotective, stress response network [49]. The earliest studies looked at the magnitude of reduced hepatic levels of aflatoxin–DNA adducts relative to a reduced tumor burden. The diminution of hepatic aflatoxin–DNA adduct levels consistently underestimated the chemopreventive efficacy [50], highlighting a quantitative disconnect between biologically effective dose markers and risk reduction. Lifetime bioassays evaluating interventions with oltipraz [37] or CDDO-Im [35] compared the initial effects on the urinary excretion of aflatoxin–N^7^–guanine to cancer reduction. In both studies, the protection against cancers was 100%, whereas the urinary biomarker, while significantly diminished, was detectable in all samples. Clearly, the notion of a threshold for DNA damage within the context of cancer risk muddles the associations between the two outcomes [51]. 

Studies focused on serum aflatoxin–albumin adducts led to similar conclusions. In a dietary intervention with the oltipraz analog 1,2-dithiole-3-thione, the overall diminutions in the levels of hepatic DNA adducts, urinary aflatoxin–N^7^–guanine, and serum aflatoxin–albumin adducts over a two-week exposure period were remarkably similar [32]. In a follow-up study, the rats were dosed with AFB_1_ daily for 5 weeks after randomization into no intervention, delayed-transient intervention (weeks 2 and 3 relative to AFB_1_), or persistent oltipraz intervention (weeks −1 to 5) groups [52]. Serial blood samples were collected from each animal at weekly intervals. The area under the curve (AUC) values for aflatoxin–albumin adducts decreased 20 and 39% in the delayed-transient and persistent oltipraz intervention groups, respectively, as compared to the value with no intervention. The total incidence of HCC dropped from 83 to 60% (*p* = 0.03) and 48% (*p* < 0.01) in these groups, highlighting a concordance between these two end points. Overall, a significant association was seen between biomarker AUC and the risk of HCC (*p* < 0.01). However, when the predictive value of aflatoxin–albumin adducts was assessed within treatment groups, there was no association between AUC and the risk of HCC (*p* = 0.56). Thus, aflatoxin–albumin adducts can be useful for monitoring population-based changes induced by interventions, such as in chemoprevention trials, but have limited utility in identifying individuals destined to develop HCC. 

***Human studies***. The seminal study on aflatoxin biomarkers and cancer risk was conducted in Shanghai, China by an international team including Professor Groopman. A prospective study of more than 18,000 men during the 1980s and 1990s revealed a statistically significant increase in the risk of liver cancer (relative risk, 3.4) when aflatoxin biomarkers were detected. For persons seropositive for HBsAg (a biomarker of hepatitis B virus (HBV) infection), the relative risk was 7.3, but, for individuals positive for both aflatoxin and HBsAg, the relative risk was 59.4, thus demonstrating a synergistic effect of these joint risk factors [53,54]. These results contributed to the International Agency for Research on Cancer classifying aflatoxins as Group 1 human carcinogens [55]. Subsequent cohort studies in Taiwan have substantially confirmed the results from the Shanghai investigation [56,57]. Notably, as in the Shanghai cohort, the liver cancer risk associated with AFB_1_ exposure was most striking among HBV carriers with detectable aflatoxin–N^7^–guanine adducts in urine. 

Using a population-based cancer registry established by the Qidong Liver Cancer Institute in 1972 and aflatoxin-specific biomarkers, Groopman and colleagues documented that the reduction in aflatoxin exposure from exceedingly high to nearly undetectable levels has likely contributed to a nearly 70% decline in age-standardized liver cancer incidence over the past 30 years despite an unchanging prevalence of HBV infection in cases [58]. A natural experiment of economic reform in the 1980s drove a rapid switch from the consumption of heavily contaminated maize to minimally, if any, contaminated rice and, subsequently, the expansion to dietary diversity. Serum levels of aflatoxin–albumin adducts in the Qidong population began declining after that switch and are now virtually undetectable [59]. The time to liver cancer diagnosis was extended as well: in 1990, the median age of diagnosis was 48 years, whilst increasing to 67 years by 2021. Perhaps aflatoxin exposures were also accelerating the time to tumors in the background of HBV-infected people. These findings have important translational public health implications, since >5 billion people in developing countries worldwide are at risk of the chronic exposure to aflatoxins through contaminated foods, especially in societies using maize as the staple food [60]. Using aflatoxin–albumin adduct biomarkers, and observations that aflatoxin exposure may affect child growth and susceptibility to infection [61], as well the liver cancer risk, served to further emphasize the public health need for the development and implementation of interventions in these populations. 

**Figure 4:**
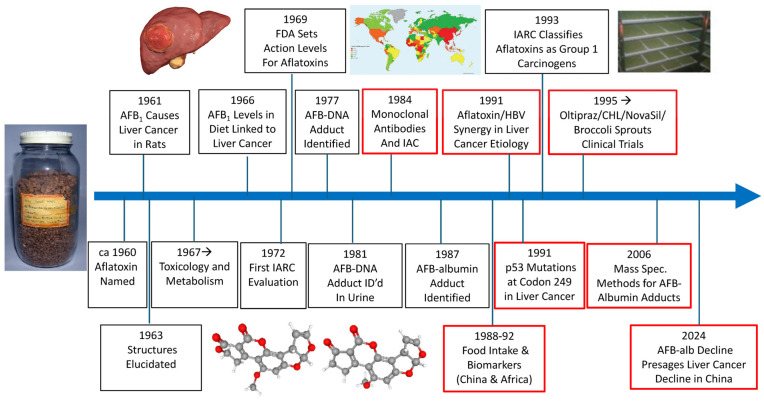
Timeline for key events in the discovery, biomarker development and molecular epidemiology, and regulation of aflatoxins. FDA, Food and Drug Administration; IARC, International Agency for Research on Cancer; IAC, immunoaffinity chromatography; CHL, chlorophyllin. Red boxes highlight seminal contributions of Professor John Groopman over the last 4 decades. Photo credits: original “Rosetta” groundnut meal used in initial characterization of aflatoxin toxicities, courtesy of John Groopman; and broccoli sprouts grown in the Qidong Liver Cancer Institute, China for the first clinical trials, courtesy of Thomas Kensler.

A timeline of key milestones in the discovery, biomarker development and molecular epidemiology, and the regulation of aflatoxins is presented in Figure 4. The key contributions of Professor Groopman to these events are highlighted.

## 4. Analytic Approaches to Aflatoxin Biomarker Development

Many different analytical methods have been applied for the quantitation of chemical adducts in biological samples. Thin-layer chromatrography, HPLC coupled with UV or fluorescence detection, and isotope-dilution mass pectrometry with ever improving detection modalities have been used in population biomonitoring. Key in biomarker development and application has been the serial improvements in sensitivity and specificity as these methodologies came online. Each methodology has unique features of specificity and sensitivity. For example, to measure a single aflatoxin metabolite, a chromatographic method can resolve mixtures of aflatoxins into individual compounds, providing that the extraction procedure does not introduce large amounts of interfering chemicals. Antibody-based methods often are more sensitive than chromatography, but immunoassays are less selective because the antibody may cross-react with multiple metabolites. Current studies using isotope-dilution and tandem mass spectrometry with liquid chromatography separation and improved detection methods have demonstrated an increase in sensitivity of at least 1000-fold over technologies previously used for the detection of aflatoxin biomarkers. Exquisite technologies are not useful, however, if they are not amenable to the high throughput needed for population-based studies at a reasonable cost. 

Biomarker measurements in biofluids have typically involved a normalization step to either urinary creatinine levels to account for variations in urine flow rates or serum albumin levels where the albumin content is measured. While the use of urinary creatinine is broadly accepted for many types of urinary biomarker measures, the use of albumin levels for normalization has been questioned recently. As discussed by Smith et al. [62], the normalization of human serum albumin adds complexity and error due to the quantitative biases of various albumin assays used across disparate studies, which are typically of much lower sensitivity than current measures of AFB_1_-lys levels. Coupled with the added time, financial costs, and sample consumption required to perform albumin normalization, there is little compelling justification for their use. 

An important advance lies in the use of both aflatoxin–DNA adduct in urine and aflatoxin-serum albumin adduct determinations to estimate daily dietary exposures. To date, the estimates using the aflatoxin–albumin adduct have been found to be of great utility for comparisons of the exposure across different populations at risk [63]. In a study in Fusui County, Guangxi, China, aflatoxin was measured in the corn porridge that was the staple food; it was calculated that 2.9% of the daily dietary exposure was converted to the aflatoxin–albumin adduct [44]. Assuming the steady-state accumulation of albumin adduct formation occurring over a 30-day time frame, the measured value by mass spectrometry of the albumin adduct level is divided by 30 to provide a daily exposure measure. A daily exposure over 1 month of 1 μg aflatoxin per day would result in a value of 17.5 pg AFB-lysine/mg albumin. The limit of detection for daily exposure by the mass spectrometry method is 14 ng AFB_1_ per day. 

## 5. Whither Next?

The concept of the ‘exposome’, as originally proposed by Professor Christopher Wild, is “composed of every exposure to which an individual is subjected from conception to death” [64]. Miller and Jones [65] have expanded this concept as “the cumulative measure of environmental influences and associated biological responses throughout the lifespan, including exposures from the environment, diet, behavior, and endogenous processes”. Aflatoxin biomarkers ably assist in the former and fall short of the later. At their best, these markers accurately reflect individual exposures of the internal dose (oxidation and conjugation metabolites and albumin adducts) and the biologically effective dose (aflatoxin–N^7^–guanine adducts). These are targeted or knowledge-based biomarkers in which the methods were optimized for each analyte. Such exposure biomarkers are powerful tools in epidemiologic studies to dampen the prevalence of exposure misclassification in case–control and cohort studies. However, their half-lives are relatively short and do not reflect the chronic, cumulative exposures throughout a lifespan, nor do they reflect the biological responses. However, because of their chemical stability in frozen biological fluids, now verified for decades, coupled with the analytical need for very minimal specimen volumes, longitudinal collections of urine or serum samples provide opportunities for retrospective reconstructions of past aflatoxin exposures and the linkage to disease outcomes at the population, if not at the individual, level. 

Analytical methods and their associated costs are now amenable to large-scale assessments of exposures from the past, present, and future (prospectively) using aflatoxin–albumin adducts. Excellent correlation between three independent methodologies has been reported [66], as has a recognition that a simple normalization to serum volume rather than additional albumin measures is superior [62]. It is conceivable to design studies to consider whether the current regulatory standards for aflatoxin levels in human diets adequately protect public health. Enough is known about the relationships between contamination levels in foodstuffs, biomarker levels in humans, and some disease outcomes, notably, liver cancer, to consider such a fundamental question. Of course, it is not simple, as multiple confounders including infections with hepatitis B or C viruses exert additive to multiplicative perturbations to the calculus for cancer, as well as the long latency between the initial exposure and disease outcome. Further, do current levels of aflatoxin exposure contribute equally to liver cancer risk in economically developed versus less developed countries? 

Biomonitoring can also address whether subpopulations exist within the U.S. or elsewhere who may experience elevated aflatoxin exposures through dietary habits involving the consumption of large amounts of food products especially prone to aflatoxin contamination. If so, does this exposure put them at a significantly elevated risk of adverse health outcomes? A cross-sectional study of aflatoxin–lysine adduct levels in samples from the National Health and Nutrition Examination Survey (NHANES) reported that about 1% of the U.S. population had detectable levels and that additional target surveillance may be warranted [67]. Such possibilities have been carried out or are being considered in Texas [68], Guatemala [69], China [70], South Asia [71,72], and Africa [62,73,74]. Can we use biomarkers to track the consequences of climate change on aflatoxin exposures and downstream health outcomes? Do regulatory changes or active interventions (dietary diversification, the introduction of stress-resistant crops or non-toxigenic forms of *Aspergillus* into farmlands, better storage practices for dietary staples, or targeted chemopreventive interventions) adequately and sustainably dampen the internal dose levels—and, presumptively, the effects on health? Aflatoxin biomarkers are essential tools for answering questions such as these.

Analytically, only a few forms of aflatoxins need to be monitored given the dominant toxicities of AFB_1_ within the family of aflatoxins. Lysine is the primary, if not sole, amino acid for adduction in albumin. However, methods are being developed for multiplexing measures of environmental exposures within albumin. Many amino acid residues are known to be sites of covalent modifications by exogenous and endogenous electrophiles (principally Cys, but also His, Tyr, Ser, Met, and Arg in addition to Lys). Thus, albumin represents a stable macromolecular platform for discerning diverse exposures through the untargeted profiling of adducts. The Rappaport [75] and Groopman [42] labs, among others, have been leaders in the application of such proteomic technologies including data deconvolution in biomarker measures of complex exposures such as air pollution that include products of exogenous (SO_2_, and benzene) and endogenous (oxidation, lipid peroxidation, glycation, and carbamylation) origin. The characterization of the exposome as envisioned by Professor Wild is becoming a reality. The inclusion of other -omic technologies, particularly metabolomics, will enable conjoined perspectives on the resultant biological responses. 

The paradigm of discovery, validation, and utilization of aflatoxin biomarkers sits at the current high point of biomarker development and the application for environmental carcinogens. Perhaps the only other chemical-specific biomarkers to reach this perch are the targeted, tobacco-specific biomarkers which arise from a very complex exposure milieu in smokers. Their use has demonstrably contributed to regulatory changes in the prohibitions of smoking in public places, the extent and impact of secondary smoke exposures, and the nature and extent of possible health risks from newer nicotine-delivery products [76]. The continued development of biomarkers reflecting exogenous agents and the resultant endogenous processes coupled to rapid, inexpensive, multiplexing platforms is needed to fulfill the goals of lifelong exposome characterizations.

## Data Availability

No new data were created or analyzed in this study. Data sharing is not applicable to this article.
